# Dogs and Cats: Reservoirs for Highly Diverse *Campylobacter jejuni* and a Potential Source of Human Exposure

**DOI:** 10.3390/ani10050838

**Published:** 2020-05-12

**Authors:** Amandine Thépault, Valérie Rose, Marilyne Queguiner, Marianne Chemaly, Katell Rivoal

**Affiliations:** 1 ANSES, Ploufragan-Plouzané-Niort Laboratory, Hygiene and Quality of Poultry and Pig Products Unit, BP53, 22440 Ploufragan, France; amandine.thepault@anses.fr (A.T.); valerie.rose@anses.fr (V.R.); maryline.queguiner@cotesdarmor.fr (M.Q.); marianne.chemaly@anses.fr (M.C.); 2 French Agency for Food, Environmental and Occupational Health & Safety, Rennes 1 University, rue du Thabor, 35000 Rennes, France

**Keywords:** *Campylobacter*, zoonosis, cat, dog

## Abstract

**Simple Summary:**

*Campylobacter* is among the most common causes of bacterial gastroenteritis and is associated with post-infectious neuropathies. This organism is part of the commensal microbiota of numerous host species, including companion animals. Molecular typing approaches have been used to attribute the source of human campylobacteriosis and showed that broilers are a major infection source, while pets are a non-negligible one (10–25% of clinical cases). Since the assessment of animal colonization by *Campylobacter* is crucial to better understand its epidemiology, we determined the *Campylobacter* carriage by dogs and cats, hereafter defined as pets, and characterized genetically *Campylobacter jejuni* (*C. jejuni)* isolates. Pets appeared to frequently carry *Campylobacter*. The comparison of genetic profiles of *C. jejuni* isolates with isolates from several animal reservoirs and clinical cases revealed an overlap between profiles. These results suggest potential pets’ contamination by livestock or inversely, as well as their potential role in *Campylobacter* transmission to humans. However, some pets’ profiles were not isolated in livestock, which suggests the existence of other sources of pet contamination by *C. jejuni* or implies that pets may constitute a reservoir for *Campylobacter* with specific profiles. Since frequent contact occurs between pets and humans, this work emphasized the potential role of pets as a source of human exposure to *Campylobacter*.

**Abstract:**

Assessing the carriage of *Campylobacter* in animal reservoirs is essential to better understand *Campylobacter* epidemiology. Here, we evaluated the prevalence of thermophilic *Campylobacter* spp. in dogs and cats, hereafter defined as pets, and characterized *Campylobacter jejuni* (*C. jejuni)* isolates to assess their genetic diversity and their potential link with isolates from other animals or human cases. During a 6-month period, 304 feces samples were collected from pets. A significantly higher prevalence of thermophilic *Campylobacter* spp. was found in dogs compared with cats, as well as in dogs ≤ 1-year-old compared with older dogs. *C. jejuni* was the predominant species found in pets, and its genomic characterization revealed a high genetic diversity. Genotypes comparison with previously characterized isolates revealed a partial overlap between *C. jejuni* isolates from pets, chicken, cattle, and clinical cases. This overlap suggests the potential role of livestock and humans in pets’ exposure to *Campylobacter*, or vice versa. The isolation of pets’ specific profiles may suggest the existence of other sources of pet contamination or imply that pets may constitute a reservoir for *Campylobacter*. Because of the proximity between humans and pets, along with their frequent carriage of *C. jejuni*, human exposure to *Campylobacter* from pets can be more important than previously thought.

## 1. Introduction

*Campylobacter* spp. are the leading cause of the main bacterial foodborne zoonosis in Europe, with about 246,571 cases reported in 2018 [1]. *Campylobacter jejuni* (*C. jejuni)* is the most frequent causative species of campylobacteriosis followed by *C. coli*. In France, these species were respectively responsible for nearly 80 and 15% of human infections, while *C. fetus*, *C. lari* and *C. upsaliensis* accounted for 4, 0.4, and 0.1% of campylobacteriosis cases that occurred between 2003 and 2010, respectively [2]. Several risk factors for human infection by *Campylobacter* spp. are related to the consumption of contaminated foodstuffs, such as chicken meat, unpasteurized milk, or untreated water. However, traveling abroad and contact with farm animals or pets also constituted risk factors for human campylobacteriosis [3,4,5]. Several source attribution studies quantifying the relative importance of animal reservoirs in campylobacteriosis identified chicken and cattle as the main source of human infection by *Campylobacter* spp. [6,7,8,9,10]. However, 1 to 25% of human campylobacteriosis are attributable to pets (including cats and dogs) in several European countries [11,12,13], where heterogeneous levels of *Campylobacter* spp. carriage by pets were highlighted [12,14,15].

Thus, cats and dogs (hereafter defined as pets) appear to be potential important sources of human infection by *Campylobacter*. To better understand the epidemiology of *Campylobacter* spp. in their natural reservoirs, several prevalence and genotyping studies have been performed in chicken, cattle, or environmental water in France. These studies revealed high rates of *Campylobacter* spp. isolation and the overlap between genetic profiles found in *C. jejuni* isolates from animal reservoirs and human campylobacteriosis, emphasizing the potential involvement of these sources in human infection [16,17,18,19]. However, no such study has been performed in pets in France, despite a possible implication of pets in transmission of *Campylobacter* spp. to humans, as previously suggested in Europe [12]. 

Therefore, this study aimed to evaluate the carriage of thermophilic *Campylobacter* spp. in pets. Then, *C. jejuni* isolates circulating in these animals were genotyped to assess their genetic diversity and their potential link with animal and clinical isolates through the comparison of their genetic profiles. For this purpose, Comparative Genomic Fingerprinting, using 40 assay genes (CGF40) [20], was used to describe *C. jejuni* isolates since this technique shows good concordance [17,20] with Multilocus Sequence Typing (MLST) [21].

## 2. Materials and Methods 

### 2.1. Collection of Feces Samples from Pets and Bacterial Analyses

In December 2014 and from April to October 2015, 304 feces samples from cats (*n =* 70) and dogs (*n =* 234) were collected on a voluntary basis (from 4 veterinarians, 2 breeders/ owners of cat and dog boarding house, and 26 private owners) in the administrative department of Côtes d’Armor in Brittany, France. Each feces sample collected was accompanied by a questionnaire informing on the age of the animal, the health status, the pet living conditions (indoor/outdoor/both), and the season of sampling. For the detection of thermophilic *Campylobacter* spp., ten grams of feces were diluted (ratio 1:10) in Bolton broth (Thermofisher Diagnostic, Dardilly, France) and homogenized for 45 s. Ten microliters of each inoculated Bolton broth were streaked onto mCCDA (modified Charcoal Cefoperazone Deoxycholate Agar) and Butzler n°2 (virion) (Thermofisher Diagnostic, Dardilly, France) plates and incubated for 72 h at 41.5 °C under microaerobic conditions. After an enrichment of the inoculated Bolton broths for 24 h at 41.5 °C, 10 µL of the mixtures were plated onto mCCDA and Butzler n°2 (virion) agar media and incubated for 48 h at 41.5 °C under microaerobic conditions. For each positive plate of each media, up to five typical *Campylobacter* colonies were subcultured onto blood agar plates for species determination by Maldi-Tof (Matrix-Assisted Laser Desorption/Ionization-Time of Flight). The isolates were stored at −70 °C in peptone broth containing 20% (v/v) glycerol, and only *C. jejuni* isolates were further characterized in this study.

### 2.2. CGF40 Typing

DNA was extracted using InstagenMatrix^®^ (Biorad, Marnes-la-Coquette, France) according the supplier recommendations. The forty accessory genes for CGF [20] were amplified according to previously published experimental conditions [17]. The CGF fingerprints were visualized using a standard gel electrophoresis containing 2% of agarose colored with GelRed^®^ (Interchim, Montluçon, France) in accordance with supplier recommendations. DNA from the reference strain NCTC11168, known to contain the 40 assay genes, was used as positive control in all assays.

The PCR results were converted into binary data corresponding to the absence (0) or the presence (1) of the marker in bacterial genomes and were stored into BioNumerics^®^ software (v6.5, Applied Maths, Belgium). A dendrogram was built using the simple matching distance coefficient and unweighted-pair group method using average linkages (UPGMA) of clustering in BioNumerics^®^ as previously described [20] and using 100% of similarity for cluster definition to describe the population structure. Then, a cut-off of 90% of similarity was used for cluster definition to compare pet *C. jejuni* isolates from this study to previously published French *C. jejuni* isolates from chicken, cattle and human campylobacteriosis [17,18,22]. Since no universal and fixed nomenclature exists regarding CGF cluster definition, the CGF 40-90% profiles of the previously published isolates were renumbered in this study.

### 2.3. Data Analysis

The *Campylobacter* status of the animals has been assessed with three main factors: (i) the kind of pets (cats or dogs), the season of sampling (spring, summer or fall) and the pet living condition (inside or outside the house or both). A multi-factor logistic regression was performed using the glm function of the R software. To assess the statistical difference between the carriage of *Campylobacter* spp. within pets according to their age, Fisher exact tests were used. In addition, the genetic diversity was assessed within *C. jejuni* isolates using the Simpson’s diversity index calculated with the online tool “Comparing Partitions” from the website http://www.comparingpartitions.info/ [23]. The calculation of the confidence intervals for clustering agreement measures at 95% was performed using the resampling technique jackknife [24].

## 3. Results

### 3.1. Campylobacter Isolation from Pet Feces and Species Identification

In this study, 304 feces samples from cats and dogs were collected: 234 samples were from dogs and 70 from cats. One hundred seventeen samples were collected in spring (89 dogs and 28 cats), 127 in summer (109 dogs and 18 cats), and 60 (36 dogs and 24 cats) in fall. According to the completed questionnaire, the age of animals ranged from 3 weeks to 17 years in dogs, and from 3 months to 15 years in cats, but no age information was collected for 5 dogs and 2 cats. Health status regarding diarrhea was also recorded, with only 3 dogs and 1 cat showed diarrhea symptoms at the sampling date. Among the 304 animals, 192 lived outdoor and indoor alternatively (156 dogs and 36 cats), 67 exclusively outdoor (65 dogs and 2 cats), 41 exclusively indoor pets (32 cats and 9 dogs), and no data were available for 4 dogs.

**Figure 1:**
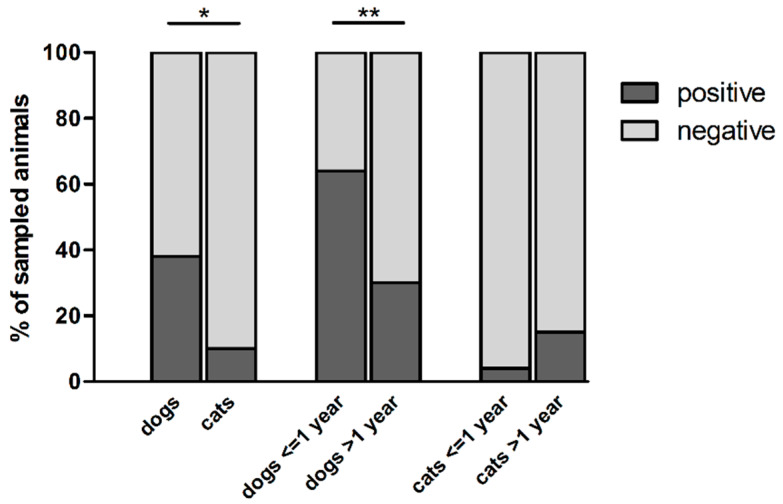
Carriage of thermophilic *Campylobacter* spp. in pets from France. * represents the significant difference between *Campylobacter* spp. carriage by dogs and cats (Fisher test, *p* < 0.0001); ** represents the significant difference between *Campylobacter* spp. carriage by dogs ≤1-year-old and older dogs (Fisher test, *p* < 0.0001).

Within the 304 pet feces samples collected, thermophilic *Campylobacter* spp. were isolated from 89 dogs and 7 cats, which correspond to a presence in 38 and 10% of the animals, respectively (Table 1; Figure 1). Dogs carried significantly more thermophilic *Campylobacter* spp. than cats (*p* < 0.0001), as well as puppies (dogs ≤ 1-year-old) compared with adult dogs (*p* < 0.0001) (Figure 1). Indeed, 63.6% (35/55) of puppies carried thermophilic *Campylobacter* spp., in comparison with 30.5% of adult dogs (53/174), while these bacteria were isolated from 20% (1/5) of dogs without age information (Table 1). No significant differences were observed in the carriage of thermophilic *Campylobacter* spp. in cats according to their age (Table 1; Figure 1). *Campylobacter* was not isolated from feces of animals showing diarrhea (*n =* 4) at the sampling date (data not shown). Because of the low number of animals showing these symptoms (4/304), its impact on *Campylobacter* carriage cannot be assessed in our study. The season of sampling, as well as the living conditions of pets, did not influence significantly *Campylobacter* carriage by pets (*p* > 0.05).

**Figure 2:**
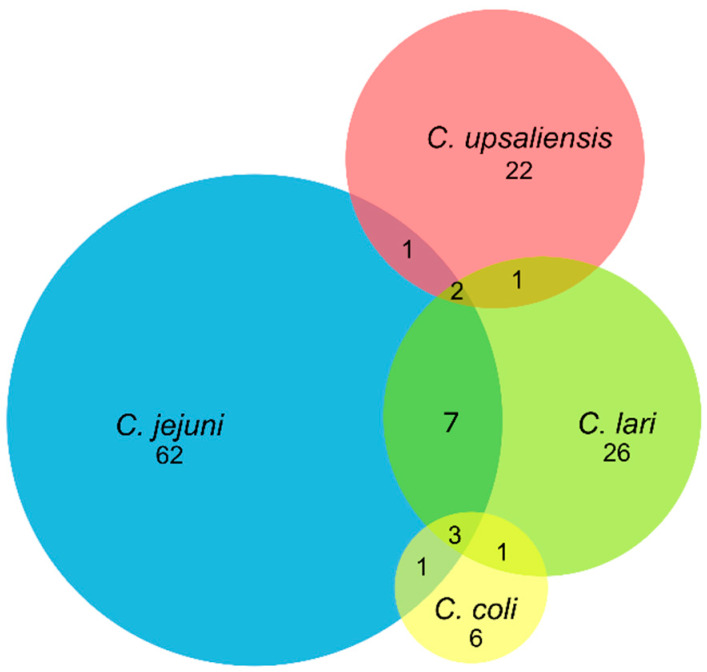
Venn diagram representing thermophilic *Campylobacter* spp. carried in pets (dogs and cats) from France. Numbers represent the number of pets carrying *Campylobacter* species. When an overlap is observed between two or more circles representing *Campylobacter* species, it means a co-infection of pets by these *Campylobacter* species.

*C. jejuni* was the main species isolated in pets, followed by *C. lari*, *C. upsaliensis*, and *C. coli* in dogs, or *C. upsaliensis* in cats (Table 1; Figure 2). *C. jejuni* was found in 64.0% (57/89) and 71.4% (5/7) of *Campylobacter*-positive dogs and cats, which corresponds to an overall *C. jejuni* prevalence of 24.4 and 7.1% in these animals, respectively (Table 1). Simultaneous carriages of several species of *Campylobacter* were observed in 16 dogs (Figure 2). Seven of them carried *C. jejuni* and *C. lari*, while carriages of *C. coli*/*C. lari*, *C. jejuni*/*C. coli*, *C. jejuni*/*C. upsaliensis*, or *C. upsaliensis*/*C. lari* were each observed in one dog. In few animals, up to three different species of thermophilic *Campylobacter* were isolated. Three dogs carried *C. jejuni*, *C. coli*, and *C. lari*, while 2 others carried *C. jejuni*, *C. lari* and *C. upsaliensis*.

### 3.2. Genotyping of C. jejuni Isolates Using Comparative Genomic Fingerprinting, Using 40 Assay Genes (CGF40)

Among 494 isolates of *C. jejuni* collected from 62 *C. jejuni*-positive feces samples, the characterization using CGF40 was performed on 455 *C. jejuni* isolates, since no growth was observed during subculture on agar plates for 39 isolates collected from 6 animals. Within the 455 genotyped isolates and based on 100% of similarity for cluster definition, 135 clusters were obtained (hereafter defined as CGF40-100% clusters). In 34 animals, from 2 to 10 different CGF40-100% clusters were detected, while 22 animals carried only one CGF40-100% cluster. Since *C. jejuni* isolates collected from the same individual animal and showing identical CGF40-100% cluster are likely to be the same strain, because of the high discriminatory power of CGF40-100% [17,20], one isolate of each CGF40-100% cluster found was kept for each animal in order to analyze the genetic structure of *C. jejuni* population.

**Figure 3:**
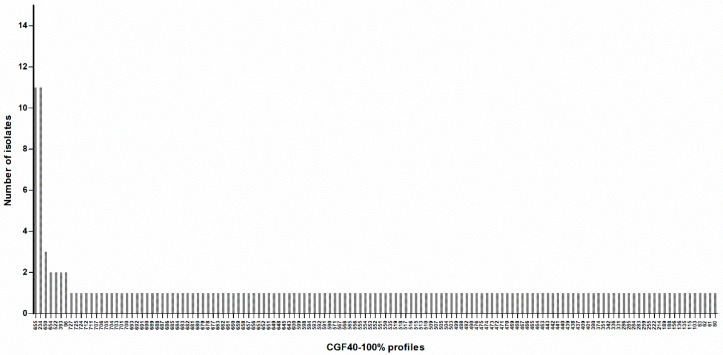
Distribution of CGF40-100% profiles within 161 *C. jejuni* isolates from cats and dogs in France. Labels on the X-axis correspond to the CGF40-100% cluster number.

Finally, this population comprised 161 *C. jejuni* isolates with 135 different CGF40-100% clusters (Figure 3; Supplementary Table S1). A Simpson’s diversity index of 0.991 (CI95% 0.984–0.998) was calculated, indicating a high genetic diversity among *C. jejuni* isolated from pets. Indeed, 79.5% of isolates (128/161) had a CGF40-100% profile found only once in the population (Figure 3; Supplementary Table S1). However, 2 profiles were predominant (profiles 234 and 655), including each 11 isolates, which represent 6.8% of the population (Figure 3; Supplementary Table S1).

**Figure 4:**
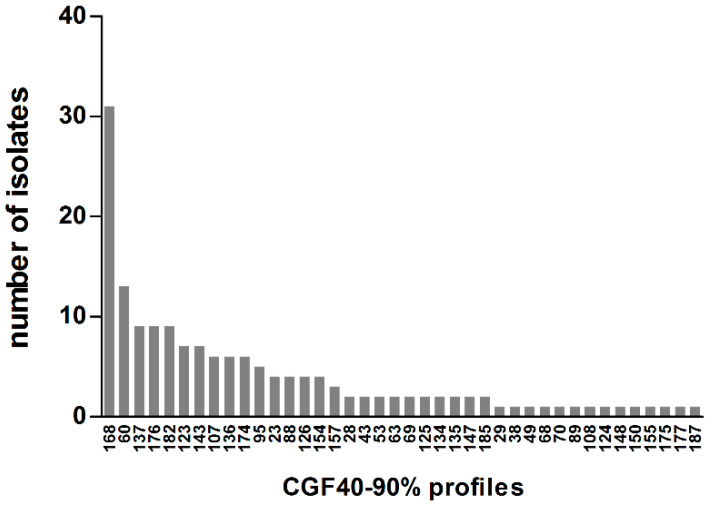
Distribution of CGF40-90% profiles within 161 *C. jejuni* isolates from cats and dogs in France. Labels on the X-axis correspond to the CGF40-90% cluster number.

To compare *C. jejuni* isolates from pets with previously characterized *C. jejuni* from chicken, cattle, or humans in France [17,18,22], CGF40 clusters were then defined on the basis of 90% of similarity and, thereafter, termed as CGF40-90% clusters. Within the 161 *C. jejuni* isolated from pets, 40 CGF40-90% clusters were obtained. By looking at the distribution of CGF40-90% clusters within the *C. jejuni* population from pets (Figure 4), five clusters, including each at least 5% of *C. jejuni* isolates, were observed and were defined as predominant.

These 5 predominant CGF40-90% clusters (clusters 168, 60, 137, 176, and 182) included 44.1% of *C. jejuni* isolates from pets, and the most prevalent cluster (168) included 19.3% of the isolates (Table 2). Three of the 5 predominant clusters in pets were found in *C. jejuni* chicken isolates, while 4 of them were also isolated from cattle and human (Table 2). The cluster 168 which is the most prevalent cluster in pets, was found in a small proportion in cattle (0.9%) and clinical cases (0.6%) but was absent within the population of chicken isolates. However, the second most prevalent cluster in pets (cluster 60) was detected in chicken, cattle isolates, as well as in clinical isolates, in which it constitutes the most prevalent CGF40–90% cluster with 13.2% of prevalence. In addition and to a lesser extent, *C. jejuni* from clusters 137 and 182 were isolated in chicken (6.2 and 3.3% of isolates, respectively), cattle (1.2 and 1.4% of isolates, respectively), and clinical cases (4.1 and 1.2% of isolates, respectively) (Table 2). Finally, no *C. jejuni* belonging to the cluster 176, which includes 5.6% of pet isolates, was collected within chicken, cattle, or human (Table 2).

## 4. Discussion

This study conducted in Brittany is the first study to our knowledge which assesses the carriage of thermotolerant *Campylobacter* spp. by pets in France and describes the genetic structure of *C. jejuni* isolates circulating in these animals. Here, we highlighted the frequent carriage of thermotolerant *Campylobacter* spp. in dogs (38%), as well as their isolation in cats (10%) to a lesser extent. Wide variations were observed in *Campylobacter* spp. prevalence in cats and dogs between several prevalence studies performed in other countries. Indeed, from 4.8 to 73% of dogs were reported to carried *Campylobacter* spp., while the prevalence ranged from 9.9 to 41.9% in cats [15,25,26,27,28,29].

Here, we also reported a higher *Campylobacter* carriage by dogs under 1-year-old (63.6%) compared with older dogs (30.5%), while no difference was observed in cats. This result emphasized the variation of dog colonization according to their age previously described in a few studies [25,30]. In addition, several epidemiological studies highlighted that young dogs are more likely to carry *Campylobacter* spp. [31], especially *C. upsaliensis* [15,27].

*C. upsaliensis* is widely described as the predominant *Campylobacter* species isolated from pets and especially dogs, while, in some studies, *C. helveticus* was described as the most prevalent *Campylobacter* species in cats [12,32,33,34]. However, in accordance with Giacomelli et al. [35] and Amar et al. [14], the species identification of *Campylobacter* spp. isolates revealed in this study the predominance of *C. jejuni* in dogs (24.4%) and cats (7.1%). In 18% of *Campylobacter*-positive dogs, co-infection with up to three different species of *Campylobacter* was also found in our study. In their study, Chaban et al. [34] reported a simultaneous carriage in healthy and diarrheic dogs with up to 7 and 12 *Campylobacter* species, respectively, while Parson, et al. [28] described simultaneous carriage with 2 different *Campylobacter* species in dogs.

However, it should be noted that since our sampling survey was conducted locally, and despite a high number of samples, our results may not be representative of the French pet population. In addition, several factors might have influenced *Campylobacter* carriage rate by pets in our study, such as technical differences in the protocol used for the detection of thermophilic *Campylobacter* spp. In our study, conducted at the scale of the administrative department of Côtes d’Armor in Brittany, the season of sampling did not appear to influence the *Campylobacter* carriage by pets. However, seasonal variations in the prevalence of *Campylobacter* spp. in pets have been previously described. Indeed, Carbonero et al. [31] described a seasonal difference in the prevalence of *C. jejuni* in dogs during the spring compared with winter, while Mohan et al. [36] reported in New-Zealand a higher prevalence of *C. jejuni* in dogs during warmer months of the year. However, this seasonal variation is debatable since other studies did not observe significant seasonal variations in *Campylobacter* spp. prevalence in dogs [32,37]. Another factor, which might have impacted the carriage rate of *Campylobacter* within pets, is their health status, even if this is debatable since contradictory results are described. In fact, Carbonero et al. [31] showed that diarrhea in pets and especially dogs are positively associated with *Campylobacter* spp., *C. jejuni* and *C. upsaliensis* carriage, while Rossi et al. [33] and Parsons et al. [27] did not observe any association between intestinal disorder and *Campylobacter* spp. carriage rates in pets. The impact of health status on *Campylobacter* carriage in dogs and cats cannot be assessed in our study because of the low number of animals showing diarrhea symptoms (4/304). Finally, the living conditions of pets might also have influenced *Campylobacter* spp. carriage. In fact, dogs having frequent contact with birds, or outdoor cats are more likely to carry *Campylobacter* spp. [15], as well as stray dogs compared with family dogs [38]. This may also be the consequences of a higher exposure of these pets to *Campylobacter* spp. through the environment or contact with birds, which are well described to frequently carry *Campylobacter* species [39,40]. In our study, performed at the scale of the administrative department of Côtes d’Armor in Brittany, the living conditions of pets (inside or outside the house or both) did not appear to influence the *Campylobacter* carriage by pets.

Pets could therefore constitute a reservoir for *Campylobacter* spp. and especially for *C. jejuni* in France. Genotyping of *C. jejuni* isolates circulating in pets using CGF40 revealed in this study an important genetic diversity, with 79.5% of *C. jejuni* isolates belonging to a CGF40-100% profile found only once in the population. The carriage of highly diverse *C. jejuni* by pets in our study was also highlighted by the isolation of several genotypes (from 1 to 10) of *C. jejuni* within the same individual, as previously described by Koene et al. [41] using another genotyping method. This high genetic diversity in *C. jejuni* isolates was also highlighted during a longitudinal study by Hald et al. [32], in which different genotypes were isolated in the same animal over the time.

Within the five main “pet CGF40-90% genotypes”, three and four of them were also isolated in chicken and cattle, respectively. This finding is in accordance with Acke et al. [42], who reported an overlap of genotypes between pets and, chicken or cattle *C. jejuni* isolates, and may indicate a potential transmission of *C. jejuni* between these animals and pets. However, the absence, or the low isolation rate, of the first and fourth main “pet CGF40-90% genotypes” in cattle and chicken may suggest the existence of other sources of pet contamination by *C. jejuni* (e.g., environmental water or wild birds) or the existence of genotypes specific to *C. jejuni* from pets, therefore implying that they may constitute a reservoir for *Campylobacter* spp. with pet-specific genotypes. This absence of pet genotypes in cattle or chicken may also be the result of rapid genetic rearrangements in the genome of *Campylobacter* hosted by pets, as hypothesized by Koene et al. [41], to explain the shedding of distinct *Campylobacter* genotypes by pets living in the same household.

Finally, an overlap of CGF40-90% genotypes was observed in pets and human isolates, as previously described using other genotyping methods [12,14,43], suggesting a potential role of pets as a source of *Campylobacter* exposure for humans. This was especially supported by the identification of some risk factors for human contamination by *Campylobacter* spp. related to pets, such as owning a dog under 1-year-old [12]. As suggested by Mughini-Gras et al. [12], the transmission to humans of *Campylobacter* carried by pets may occur either directly by contact or by the household and the immediate environment contamination since these animals may eventually act as reservoirs for *Campylobacter*. However, the overlap between genotypes in pets and humans may also indicate a common infection of pets and humans by another source, such as cattle or chicken, which also overlapped with humans and pets, or a potential role of human as a source of pets contamination by *Campylobacter* spp. [12].

## 5. Conclusions

Pets constitute an important reservoir for *Campylobacter* spp. in our sampling area, and *C. jejuni* was the most frequent species isolated, while co-infections of individual animals with several species of *Campylobacter* were reported. Dogs appeared to carry more *Campylobacter* spp. than cats, as well as young dogs compared with dogs older than 1 year. The genetic characterization of *C. jejuni* isolates using CGF40 highlighted the high genetic diversity within the isolates population and the co-infection of individual animals by several genotypes of *C. jejuni*. Then, the overlap between pet and chicken or cattle isolates indicated the potential role of the livestock as a source of contamination for pets or inversely, while the overlap between isolates from pets and humans suggested a potential role of pets in human contamination by *C. jejuni*, or vice versa. Because of low attribution of human campylobacteriosis to pets in source attribution studies, the risk for human health is considered low. Nevertheless, because of the proximity between pets and humans, and the frequent carriage of *C. jejuni* by these animals in our study, the exposure of human to *Campylobacter* from pets can be more important than thought, and pets can constitute a significant source of human exposure to *Campylobacter*. Further analyses will be necessary to assess with accuracy the implication of pets in human campylobacteriosis.

## Figures and Tables

**Table 1:** Prevalence of thermophilic *Campylobacter* species from dogs and cats feces samples in France.

Bacterial Species Isolated	Dogs								Cats							
	Total (*n =* 234)		≤1 year (*n =* 55)		>1 year ( *n*= 174)		N/A (*n =* 5)		Total (*n =* 70)		≤1 year (*n =* 26)		>1 year (*n =* 42)		N/A (*n =* 2)	
	*n*	%	*n*	%	*n*	%	*n*	%	*n*	%	*n*	%	*n*	%	*n*	%
*Campylobacter* spp.	89	38.0 *	35	63.6 **	53	30.5	1	20.0	7	10.0	1	3.8	6	14.3	0	0
*C. jejuni*	57	24.4	24	43.6	32	18.4	1	20.0	5	7.1	1	3.8	4	9.5	0	0
*C. coli*	6	2.6	4	7.3	2	1.1	0	0	0	0	0	0	0	0	0	0
*C. lari*	26	11.1	12	21.8	14	8.0	0	0	0	0	0	0	0	0	0	0
*C. uspaliensis*	21	9.0	8	14.5	13	7.5	0	0	1	1.4	0	0	1	2.4	0	0
*Campylobacter* species not identified	0	0	0	0	0	0	0	0	1	1.4	0	0	1	2.4	0	0

* Significantly higher prevalence of *Campylobacter* spp. in dogs compared with cats (Fisher test, *p* < 0.001). ** Significantly higher prevalence of *Campylobacter* spp. in young dogs compared with older dogs (Fisher test, *p* < 0.001). Note: Co-infection with two or three *Campylobacter* species was observed within 16 dogs.

**Table 2:** Presence of the 5 main CGF40-90% clusters of *C. jejuni* from pets, among chicken, cattle, or human isolates in France.

CGF40-90% Cluster	Pets	Chicken	Cattle	Human
168	31/161 (19.3%)	0/644 (0%)	6/649 (0.9%)	3/514 (0.6%)
60	13/161 (8.1%)	51/644 (8.0%)	66/649 (10.2%)	68/514 (13.2%)
137	9/161 (5.6%)	40/644 (6.2%)	8/649 (1.2%)	21/514 (4.1%)
176	9/161 (5.6%)	0/644 (0%)	0/649 (0%)	0/514 (0%)
182	9/161 (5.6%)	21/644 (3.3%)	9/649 (1.4%)	6/514 (1.2%)

## Supplementary Materials

The following are available online at https://www.mdpi.com/2076-2615/10/5/838/s1, Table S1: Genetic characteristics of *C. jejuni* isolates collected from pets in France.
